# Supplementary material for: MetaRibo-Seq measures translation in microbiomes
Source: Nat Commun. 2020 Jun 29;11:3268. doi: 10.1038/s41467-020-17081-z (PMC7324362; doi:10.1038/s41467-020-17081-z)
Supplement: Supplementary file 10 — Supplementary Data 7 [file 41467_2020_17081_MOESM10_ESM.zip › File2/Confidence_High_Taxonomy/394876_out.krona.html]

Javascript must be enabled to view this page.

members
magnitude
magnitudeUnassigned
count
unassigned
taxon
rank

394876\_out

136

136
superkingdom
2

136
phylum
976

136
class
200643

171549
136

SRS016989\_contig\_number\_3590SRS049900\_contig\_number\_contig-100\_1023.122041SRS049959\_contig\_number\_14530SRS076929\_contig\_number\_contig-100\_1067.136333SRS143085\_contig\_number\_4512SRS144297\_contig\_number\_6672
order
6

815
family
13

816
genus
13

162156

SRS020622\_contig\_number\_1234
1
species

821
species

SRS019267\_contig\_number\_820
1

1739517

SRS063040\_contig\_number\_13617
1
species

species

SRS147377\_contig\_number\_8968
1
820

species
1

SRS076804\_contig\_number\_10849
817

1

SRS011084\_contig\_number\_14393
species
469589

246787

SRS062701\_contig\_number\_5874SRS077086\_contig\_number\_3671SRS1055038\_contig\_number\_565SRS145497\_contig\_number\_11050
4
species

818
1

SRS065504\_contig\_number\_1090
species

species
1

SRS893252\_contig\_number\_16739
2292944

species

SRS100021\_contig\_number\_5449
1
46506

171551
7
family

836
genus
7


SRS015190\_contig\_number\_3303SRS015264\_contig\_number\_17557SRS019445\_contig\_number\_20767SRS043411\_contig\_number\_12707SRS050299\_contig\_number\_9981SRS104975\_contig\_number\_17860SRS147346\_contig\_number\_21843
7
species
658663

108
family
2005525

genus
23
375288
108

SRS014287\_contig\_number\_11758SRS018351\_contig\_number\_19185SRS018575\_contig\_number\_2566SRS019068\_contig\_number\_58447SRS019808\_contig\_number\_2835SRS019968\_contig\_number\_12739SRS043667\_contig\_number\_3355SRS045244\_contig\_number\_6765SRS047014\_contig\_number\_33711SRS048981\_contig\_number\_contig-100\_1891.30791SRS050941\_contig\_number\_contig-100\_172.114503SRS051031\_contig\_number\_contig-100\_3370.151956SRS056519\_contig\_number\_15322SRS058770\_contig\_number\_23406SRS063370\_contig\_number\_4388SRS075021\_contig\_number\_861SRS075078\_contig\_number\_contig-100\_62.187415SRS075878\_contig\_number\_11269SRS077294\_contig\_number\_11486SRS098717\_contig\_number\_2399SRS1041090\_contig\_number\_5197SRS148721\_contig\_number\_34889SRS148970\_contig\_number\_1595

823

SRS012969\_contig\_number\_21155SRS013098\_contig\_number\_15053SRS013800\_contig\_number\_14774SRS014235\_contig\_number\_28033SRS014412\_contig\_number\_7657SRS014948\_contig\_number\_12766SRS015217\_contig\_number\_30086SRS015579\_contig\_number\_18203SRS015960\_contig\_number\_6768SRS016056\_contig\_number\_2168SRS016095\_contig\_number\_21030SRS016267\_contig\_number\_8285SRS016335\_contig\_number\_15148SRS017191\_contig\_number\_20315SRS017307\_contig\_number\_16287SRS017701\_contig\_number\_16017SRS018541\_contig\_number\_1781SRS018836\_contig\_number\_25931SRS018888\_contig\_number\_1159SRS019178\_contig\_number\_2460SRS019601\_contig\_number\_4817SRS019685\_contig\_number\_36301SRS019787\_contig\_number\_8890SRS023526\_contig\_number\_23486SRS023583\_contig\_number\_11096SRS023829\_contig\_number\_contig-100\_34.34SRS024132\_contig\_number\_41929SRS043701\_contig\_number\_10695SRS043768\_contig\_number\_24622SRS045195\_contig\_number\_1167SRS045826\_contig\_number\_14941SRS046712\_contig\_number\_892SRS048164\_contig\_number\_15089SRS049712\_contig\_number\_11707SRS050422\_contig\_number\_10771SRS050752\_contig\_number\_24898SRS051882\_contig\_number\_10159SRS052027\_contig\_number\_4251SRS053398\_contig\_number\_24090SRS056259\_contig\_number\_16732SRS056273\_contig\_number\_17048SRS058145\_contig\_number\_3804SRS064645\_contig\_number\_343SRS064973\_contig\_number\_1584SRS065397\_contig\_number\_13358SRS075398\_contig\_number\_2191SRS077454\_contig\_number\_12003SRS077849\_contig\_number\_29006SRS078419\_contig\_number\_30587SRS097920\_contig\_number\_26431SRS103987\_contig\_number\_5444SRS104036\_contig\_number\_6941SRS1041036\_contig\_number\_1654SRS1041091\_contig\_number\_10212SRS1041095\_contig\_number\_1080SRS1041138\_contig\_number\_11149SRS1041142\_contig\_number\_1253SRS104311\_contig\_number\_9183SRS142599\_contig\_number\_30841SRS142890\_contig\_number\_37237SRS143148\_contig\_number\_12324SRS143342\_contig\_number\_14672SRS143523\_contig\_number\_21247SRS143876\_contig\_number\_35552SRS144183\_contig\_number\_11048SRS144506\_contig\_number\_52680SRS144603\_contig\_number\_11807SRS146764\_contig\_number\_9668SRS146813\_contig\_number\_17047SRS147272\_contig\_number\_965SRS148196\_contig\_number\_contig-100\_11.227025SRS148511\_contig\_number\_4363SRS148784\_contig\_number\_18466SRS148874\_contig\_number\_15306SRS893259\_contig\_number\_1986SRS893285\_contig\_number\_945SRS893288\_contig\_number\_3836
77
species

1947048
species
2

SRS097958\_contig\_number\_21787SRS971276\_contig\_number\_8342

3

SRS046369\_contig\_number\_9260SRS052078\_contig\_number\_2379SRS147614\_contig\_number\_7619
species
46503

563193
3

SRS076976\_contig\_number\_15655SRS1055076\_contig\_number\_10196SRS893342\_contig\_number\_8192
species

1853231
family
1

1
genus
283168

28118
species

SRS015431\_contig\_number\_20644
1

1
family
171550

genus
1
239759


SRS053649\_contig\_number\_10762
1
species
28117
